# The mutations on the envelope glycoprotein D contribute to the enhanced neurotropism of the pseudorabies virus variant

**DOI:** 10.1016/j.jbc.2023.105347

**Published:** 2023-10-12

**Authors:** Hongxia Wu, Hansong Qi, Bing Wang, Mingzhi Li, Liang Qu, Su Li, Yuzi Luo, Lian-Feng Li, Guang-Lai Zheng, Hua-Ji Qiu, Yuan Sun

**Affiliations:** State Key Laboratory for Animal Disease Control and Prevention, Harbin Veterinary Research Institute, Chinese Academy of Agricultural Sciences, Harbin, China

**Keywords:** pseudorabies virus, variant strain, envelope protein, neurotropism, virulence

## Abstract

The pseudorabies virus (PRV) TJ strain, a variant of PRV, induces more severe neurological symptoms and higher mortality in piglets and mice than the PRV SC strain isolated in 1980. However, the mechanism underlying responsible for the discrepancy in virulence between these strains remains unclear. Our study investigated the differences in neurotropism between PRV TJ and PRV SC using both *in vitro* and *in vivo* models. We discovered that PRV TJ enters neural cells more efficiently than PRV SC. Furthermore, we found that PRV TJ has indistinguishable genomic DNA replication capability and axonal retrograde transport dynamics compared to the PRV SC. To gain deeper insights into the mechanisms underlying these differences, we constructed gene-interchanged chimeric virus constructs and assessed the affinity between envelope glycoprotein B, C, and D (gD) and corresponding receptors. Our findings confirmed that mutations in these envelope proteins, particularly gD, significantly contributed to the heightened attachment and penetration capabilities of PRV TJ. Our study revealed the critical importance of the gD^ΔR278/P279^ and gD^V338A^ in facilitating viral invasion. Furthermore, our observations indicated that mutations in envelope proteins have a more significant impact on viral invasion than on virulence in the mouse model. Our findings provide valuable insights into the roles of natural mutations on the PRV envelope glycoproteins in cell tropism, which sheds light on the relationship between cell tropism and clinical symptoms and offers clues about viral evolution.

Pseudorabies virus (PRV) is a highly contagious virus that infects pigs and causes significant economic losses in the swine industry. While PRV infections in older pigs typically cause respiratory and reproductive disorders, they are often proved fatal in neonatal piglets (1). Before 2011, pseudorabies occurred sporadically in China (2). However, since then, it has reemerged in many pig farms, and several PRV variants have been isolated during the epidemic (3). One of the most concerning variants is the PRV TJ strain (isolated in 2012), which exhibits increased virulence and higher mortality in pigs (4). Additionally, a PRV variant strain, hSD-1/2019, was isolated from a patient displaying clinical signs (5). The emergence of these highly virulent PRV strains highlights the need for enhanced surveillance, diagnosis, and control measures to prevent further spread and protect the health of both animals and humans.

Based on the phylogenic analysis of the gC nucleotide sequences, PRV SC (isolated in 1980) and Bartha-K61 belong to genotype I, while PRV TJ and PRV hSD-1/2019 belong to genotype II (6). Previous studies have shown that mutations in the gB, gC, and gD proteins are responsible for PRV variants evading Bartha-K61 vaccine-induced immunity (7, 8). These mutations can alter both the immunogenicity and pathogenicity of PRV variants. However, the mechanisms underlying the enhanced virulence of PRV TJ remain unknown. Further research is needed to identify the specific mutations responsible for the increased pathogenicity of PRV TJ.

PRV belongs to the genus *Varicellovirus* of the subfamily *Alphaherpesvirinae* within the family Herpesviridae (9). PRV can infect newborn piglets through various routes, including the eye, mouth, nose, and skin lesions. After infecting epithelial cells, the virus can disseminate to peripheral neuron system (PNS) by invading sensory and motor nerves eventually reaching the central nervous system (CNS) (10, 11). In older pigs, PRV can establish lifelong latent infections in the PNS and can reactivate, occasionally spreading from the CNS back to the PNS (12). Mice infected with the PRV Becker strain *via* the limb inoculation, an abundant infectious virus in the dorsal root ganglion (DRG) and spinal cord, were detected, but only a small amount is present in the brain before death. PRV can also disseminate to internal organs, including the lungs, kidneys, and uterus, which can lead to organ damage in mice. Moreover, PRV infection in mice can also cause severe itch and neurological disorders (13).

Several envelope glycoproteins of PRV, including gB, gD, gH, and gL, are crucial for the virus to penetrate neurons. Additionally, other glycoproteins, such as gC, gM, and gK, can modulate the attachment and fusion of PRV (14, 15, 16, 17). During the attachment phase, gC and gB bind the heparan sulfate proteoglycans on the host cell surface (18, 19). Simultaneously, gD facilitates the attachment by binding to the nectin-1 receptor (20), which is essential for PRV entry. The gB protein serves as the viral fusion protein and plays an essential role during viral entry into the host cell (21). The progeny viruses of the gD-deleted PRV, which are released from non-gD–complemented cells, can attach to cells but remain noninfectious (22, 23). Nectin-1 has also been identified as the primary receptor for herpes simplex virus-1 (HSV-1) penetration into mouse sensory neurons (24). The gH and gL heterodimer binds directly to integrin *ɑ*V*β*3, and gB binds to a lipid raft–associated receptor (25, 26, 27). Meanwhile, the PRV viral particles of the gL-null mutant could not penetrate and propagate in the trigeminal, sympathetic, and parasympathetic nerves. On the other hand, the absence of gC and gK only slows the invasion of the nervous system (15). Deletions of gE and gI reduce the spread of viral particles between neurons (16).

In addition to the nectin-1 receptor interacted with gD, the paired immunoglobulin-like type 2 receptors PILRα and PILRβ can bind to the gB of PRV, all these receptors facilitate viral entry into host cells (28, 29, 30). The binding process is mediated by two crucial residues, Thr-53 and Thr-480, and mutations in these residues prevent both HSV-1 and PRV from entering the CHO cells expressing PILRα (29). Similarly, mutations in gD proteins of both HSV and PRV have been identified to enhance their affinity for their respective receptors. For example, the deletion of the C terminus to 285 residues of HSV-1 gD increases its affinity for herpes virus entry mediator and nectin-1 receptors by 50- to 100-fold. Additionally, the mutation at 294th residue of HSV-1 gD enhances the binding efficiency to these receptors (31). These findings highlight the critical roles of both gB and gD in PRV entry into cells and underscore the importance of specific residues within these proteins in mediating receptor binding and viral entry processes.

The PRV SC and TJ strains exhibit dissimilarities in 43 viral proteins and have been categorized into distinct clades based on the evolutionary analysis of their gC sequences. It has been reported that pigs infected with PRV TJ displayed more severe neurological symptoms than those infected with PRV SC (4). In this study, we aimed to compare the spreading of PRV TJ and SC *in vitro* and *in vivo* and identify the specific mutations in the gB, gC, and gD that are responsible for the enhanced pathogenicity and neurological signs observed in PRV TJ. The findings of this study hold broad significance as they establish foundational principles for developing effective vaccines to prevent PRV variant infections.

## Results

### The viral loads in the nerve tissues of the PRV TJ–infected mice are higher than those of the PRV SC–infected mice

**Figure 1:**
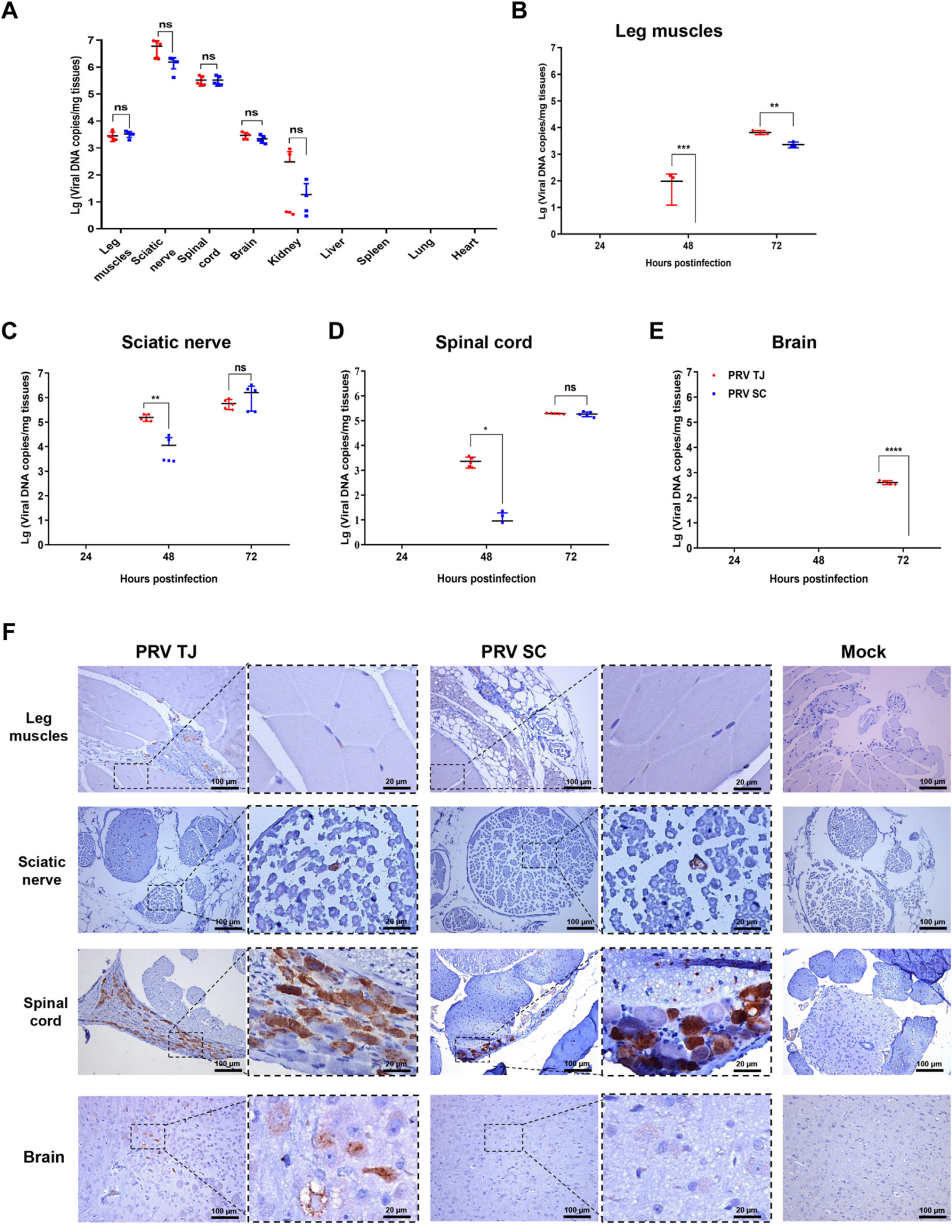
**Viral loads in the tissues of the mice infected with the PRV TJ or SC strain.** The mice were either mock-infected or infected with 10^3^ PFU of PRV TJ or SC strains, and the tissue samples were collected every day after infection until death. Legs and sciatic nerves on the inoculated side, spinal columns, and whole brains were isolated for total DNA extraction and histopathological examinations. Viral DNA copies per milligram of tissues were measured by qPCR using specific primers targeting the *gI* gene of PRV. *A*, the viral loads in the tissues of deceased mice, including the leg, sciatic nerve, spinal cord, brain, heart, liver, spleen, lungs, and kidney were detected. *B*–*E*, the viral loads in the legs, sciatic nerves, spinal cords, and brain tissues collected at 24, 48, and 72 hpi, respectively. *F*, representative immunohistochemistry (IHC) images of the legs, sciatic nerves, spinal columns, and brains collected at 72 hpi (scale bar represents 100 μm). The gB-positive region was marked by the *black broken line* (scale bar represents 20 μm). The data represents the average viral DNA copies and ± SD for the five replicates per group; ns, not significant; ∗*p* < 0.05; ∗∗*p* < 0.01; ∗∗∗*p* < 0.001; ∗∗∗∗*p* < 0.0001. gB, glycoprotein B; PFU, plaque-forming unit; PRV, pseudorabies virus; qPCR, quantitative PCR.

In comparison to PRV SC, the PRV TJ is well-documented for its heightened pathogenicity and causing more severe neurological disorders in both mice and pigs (2, 32). In this study, six-week-old specific pathogen-free (SPF) mice (*n* = 5) were intramuscularly (i.m.) infected with 10^3^ plaque-forming units (PFU) of PRV TJ or SC in the left hind leg. The mice infected with PRV TJ displayed intense itching, spun around continuously, and frequently scratched at 59.4 hours postinfection (hpi), whereas those infected with PRV SC displayed similar symptoms at 68.8 hpi, and the average clinical symptom scores of both PRV TJ and SC were 3 (Table 1). To investigate the possible reasons for the different clinical signs, we collected tissues (legs, sciatic nerves on the inoculated side, spinal columns, brain, heart, liver, spleen, lungs, and kidneys) from the infected and mock mice to quantify the viral genome copies. Viral DNA was detected only in the infected mice's legs, kidneys, and neural tissues but not in the tissues of the heart, liver, spleen, and lungs. Furthermore, there was no significant difference in the viral genome copies between the PRV TJ- and SC-infected groups in the examined tissues at the time of death (Fig. 1*A*).

**Table 1:** The outcome of the mice infected with four PRV strains

Groups	Amounts	Doses (PFU)	Clinical signs	Morbidity (mean onset time, hpi)	Mortality (mean death time, hpi)	LD_50_ (PFU)
PRV TJ	5	10^3^	3.00 ± 0.00	5/5(59.40 ± 2.30)^a^	5/5(71.00 ± 5.20)	
	5	10^2^	2.60 ± 0.55	3/5(64.67 ± 3.51)	3/5(87.33 ± 10.21)	10^1.5^
	5	10^1^	2.00 ± 1.00	2/5(90.00 ± 15.56)	2/5(102.50 ± 16.26)	
PRV SC	5	10^3^	3.00 ± 0.00	5/5(68.80 ± 5.36)^a^	5/5(76.60 ± 7.60)	
	5	10^2^	2.20 ± 1.11	3/5(74.67 ± 7.51)	3/5(105 ± 13.45)	10^1.83^
	5	10^1^	1.00 ± 1.25	0/5	0/5	
rPRVTJ-SCgBgCgD	5	10^3^	3.00 ± 0.00	5/5(65.40 ± 4.10)	5/5(78.00 ± 7.48)	
	5	10^2^	2.60 ± 0.89	3/5(80.25 ± 6.40)^b^	3/5(116.67 ± 18.72)^b^	10^1.83^
	5	10^1^	0.80 ± 1.30	0/5	0/5	
rPRVSC-TJgBgCgD	5	10^3^	3.00 ± 0.00	5/5(60.40 ± 3.21)	5/5(72.40 ± 8.91)	
	5	10^2^	2.20 ± 1.10	4/5(66.00 ± 5.00)^b^	4/5(88.50 ± 10.34)^b^	10^1.5^
	5	10^1^	0.40 ± 0.55	1/5(89.00 ± 0.00)	1/5(92.00 ± 0.00)	
DMEM	5	100 μL	0	0/5	0/5	

Mice were injected intramuscularly with three doses of each PRV strain shown in the table, with five mice in each group. Itch onset and death were recorded. Clinical symptoms were scored in four degrees: 0 (no clinical symptom), 1 (mild symptoms, including subtle neurological symptoms, untidy hair, and minor depression), 2 (common symptoms, such as pruritus, rolling, and scratching the injection site, and 3 (severe symptoms, such as severe pruritus, self-mutilate even acute death). -, death. The results are presented as the mean ± SD (*n* = 5) of the total value of four parameters, onset time, and death time.

a *p* < 0.01.

b *p* < 0.05.

The average time of death for the mice infected with 10^3^ PFU PRV TJ and SC strains was 71.00 and 76.60 hpi, respectively (Table 1). The viral DNA copies in the leg muscle tissues of the PRV TJ–infected mice exceeded those of PRV SC at 48 and 72 hpi (Fig. 1*B*). After PRV infection, viral particles entered neurons within the muscle, then spread in PNS, and eventually reached the CNS. The amount of viral DNA in the sciatic nerve on the injection side and spine of the PRV TJ–infected group surpassed those of the SC strain at 48 hpi, eventually equalizing at 72 hpi (Fig. 1, *C* and *D*). Remarkably, viral DNA of PRV TJ, but not PRV SC, was detected in the brain at 72 hpi (Fig. 1*E*).

**Fig. S1:**
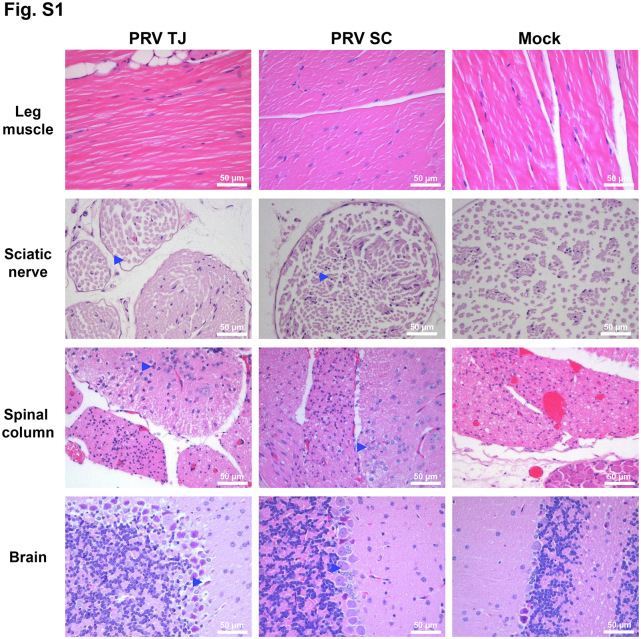
H&E images of the legs, sciatic nerves, spinal columns, and brains isolated at 72 hpi (scale bar represents 50 μm).

Immunohistochemistry (IHC) (Fig. 1*F*) and histopathological examinations (Fig. S1) were performed on nerve tissues harvested at 3 days postinfection to visualize the distribution of PRV antigens and the pathological lesions of the examined tissues. PRV gB antigens presented in the muscle cells and sciatic nerve fiber infected with PRV TJ and PRV SC strains. Furthermore, PRV gB antigens were detected in the DRG neurons of both the PRV TJ and PRV SC groups, located in proximity to the spinal cord, but not in the spinal cord. gB antigen was detected only in the brain of PRV TJ–infected mouse, but not in the PRV SC and mock group at 72 hpi.

Histopathological analysis (Fig. S1) revealed that the fibers of the sciatic nerve were seriously atrophied and mildly demyelinated, while polymorphonuclear leukocytes infiltrated widely in the gray matter, and telangiectasia and congestion were observed throughout the spinal cord in both PRV TJ- and SC-infected mice. Purkinje cells in the cerebellum of PRV TJ–infected mice showed degenerated or shrinking changes, whereas no lesions were observed in the cerebellum of the PRV SC–infected mice. These findings collectively suggest that PRV TJ exhibited higher viral loads and more severe neural tissue lesions in comparison to PRV SC.

### The PRV TJ strain is more productive than the PRV SC strain in primary neurons and N2a cells

To investigate the underlying reason for the elevated viral loads observed in the PRV TJ–infected mice, we isolated primary DRG neurons from neonatal mice and cultured them in a microfluidic system. This system physically separates the cell body (S compartment) and axon terminal (N compartment) of neuron into two chambers without allowing for medium interchange between them.

**Figure 2:**
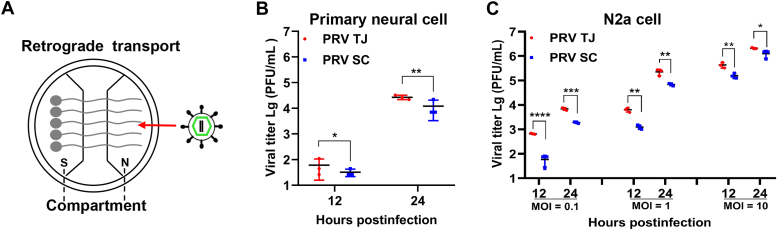
**Viral growth of the PRV TJ and SC strains in primary neurons and N2a cells.***A*, schematic of microfluidics equipment. *B*, the neurons were seeded in the S compartment of the microfluidics equipment and cultured for 4 days until the neuron's axon extended through the channel in the middle to the N compartment. Then, a total of 10^6^ PFU of either PRV TJ or SC strains were inoculated into the N compartment, and the viral titers in the S compartment of the microfluidics equipment were measured at 12 and 24 hpi. *C*, the viral proliferation of the PRV TJ and SC strains in N2a cells. The two PRV strains were inoculated at an MOI of 0.1, 1, and 10. Unbound viruses were removed at one hpi, and the cell supernatants were collected at 12 and 24 hpi to measure the viral titer by plaque assay. The bars represent the means ± SD for three independent experiments; ns, not significant; ∗*p*< 0.05; ∗∗*p* < 0.01; ∗∗∗*p* < 0.001; ∗∗∗∗*p* < 0.0001. MOI, multiplicity of infection; PFU, plaque-forming unit; PRV, pseudorabies virus.

The N compartment of the microfluidic system was inoculated with either the PRV TJ or SC strains (Fig. 2*A*), and the viral loads in the medium of the S compartment was analyzed at 12 and 24 hpi. The results showed that the virus titer in the PRV TJ group was significantly higher than that in the PRV SC group at both 12 and 24 hpi (Fig. 2*B*). The N2a cells possess the characteristics of neural cells but devoid of long axons. The N2a cells were infected with the two PRV strains at varying multiplicities of infection (MOIs = 0.1, 1, or 10), and unbound viruses were removed at one hpi. The results indicated that the titer of PRV TJ in each infected group consistently surpassed that of PRV SC at both 12 and 24 hpi (Fig. 2*C*).

### The PRV TJ strain enters neurons more efficiently than the PRV SC strain

**Figure 3:**
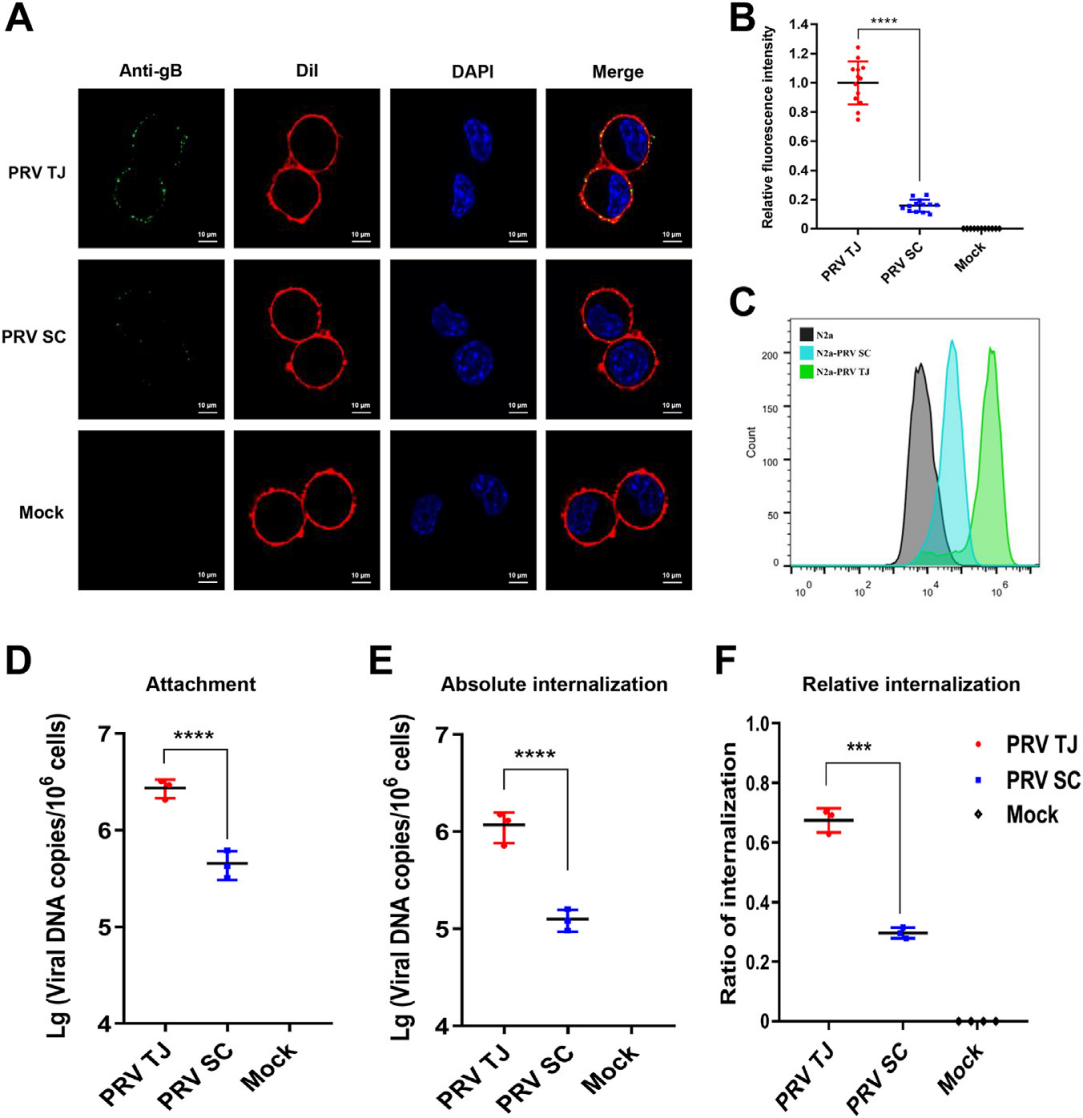
**The efficiency of PRV TJ or SC entry into N2a cells.** N2a cells were incubated with the PRV TJ and SC strains at 4 °C for 2 h, then washed three times with ice-cold PBS to remove the unbound PRVs, fixed with polyformaldehyde solution for IFA and FCM assays; moreover, total DNA was extracted from unfixed cells for qPCR assay. Viral particles attached to N2a cells infected with the PRV TJ and SC strains at an MOI of 100, 100, and 10 were then analyzed using IFA, FCM, and qPCR assays. *A*, confocal fluorescence imaging for cells bound with viral particles (scale bar represents 10 μm). The cytomembrane of N2a cells was labeled with DiI (*red*), and PRV viral particles were stained with an anti-gB primary antibody and Alexa 488–conjugated secondary antibody (*green*). The nuclei were stained with DAPI (*blue*). *B*, the average green fluorescence intensity per cell bound with PRV TJ or SC particles is based on confocal fluorescence images. *C*, FCM assay of N2a cells bound with PRV TJ or SC. PRV gB was labeled with Alexa 488 (FITC). The normal cells were shown as controls (*blank lines*), and the PRV-infected cells were marked with *green* (PRV TJ) and *blue* (PRV SC) lines. To determine PRV internalization, cells were transferred from 4 °C to 37 °C for 1 h and treated with 200 μl of 0.25% trypsin-EDTA at 4 °C for 15 min to remove the virions remaining outside the cells. Extracellular and intracellular viral DNA copies were measured by qPCR. (*D*) Attachment, (*E*) absolute internalization, (*F*) relative internalization. The bars represent the means ± SD for three independent experiments; ns, not significant; ∗∗∗*p* < 0.001; ∗∗∗∗*p* < 0.0001. FCM, flow cytometry; gB, glycoprotein B; IFA, immunofluorescence assay; MOI, multiplicity of infection; PRV, pseudorabies virus; qPCR, quantitative PCR.

We hypothesized that differences in the viral invasion process may contribute to the disparity in viral proliferation between the two PRV strains. To investigate this, we analyzed the attachment and internalization of the virus. Initially, N2a cells were infected with each PRV strain and incubated at 4 °C for 1 h. Subsequently, the cells were washed three times with PBS, and the attachment capabilities were evaluated using indirect immunofluorescence assay, flow cytometry, and quantitative PCR (qPCR). Numerous green fluorescent dots were observed outside the cell membrane of both PRV TJ- and SC-infected N2a cells (Fig. 3*A*).

However, the fluorescence intensity on the surface of the PRV TJ–infected N2a cells exceeded that of the SC-infected cells (Fig. 3*B*). Flow cytometry analysis showed that the fluorescence intensity of the PRV TJ–infected cells was significantly higher than that of the PRV SC strain, and the positive rates of the PRV TJ and PRV SC groups reached 95% and 20% in N2a cells, respectively (Fig. 3*C*). Furthermore, the average viral DNA copies of PRV TJ and SC strains attached to cells were 2.27 × 10^6^ and 0.53 × 10^6^, respectively. This observation indicated that the viral DNA copies of the PRV TJ–attached N2a cells were four-fold higher than those of the PRV SC–attached cells (Fig. 3*D*). In essence, these results suggested that the adsorption efficiency of the PRV TJ strain was significantly higher than that of the SC strain.

The internalization efficiency of the PRV TJ and SC strains into the N2a cells was also analyzed by quantifying the viral DNA copies of PRV particles attached to the cell membrane and within the cytoplasm using qPCR and calculating absolute and relative internalization values. The results revealed that the average viral DNA copies per 10^6^ cells from PRV TJ group (1.53 × 10^6^) were ten-fold higher than those of the PRV SC group (1.80 × 10^5^) within the cytoplasm of the N2a cells (Fig. 3*E*). Moreover, the relative internalization efficiencies of PRV TJ and PRV SC in the N2a cells were 67.4% and 29.6%, respectively (Fig. 3*F*).

### The PRV TJ and SC strains display comparable axonal retrograde mobility

The retrograde axonal mobility of the two PRV strains was compared by constructing recombinant viruses with the enhanced GFP (EGFP) gene fused at the N terminus of the UL36 gene, enabling the visualization of viral particle movement. The growth kinetics and morphology of these recombinant viruses closely resembled those of the parental viruses (33, 34).

**Figure 4:**
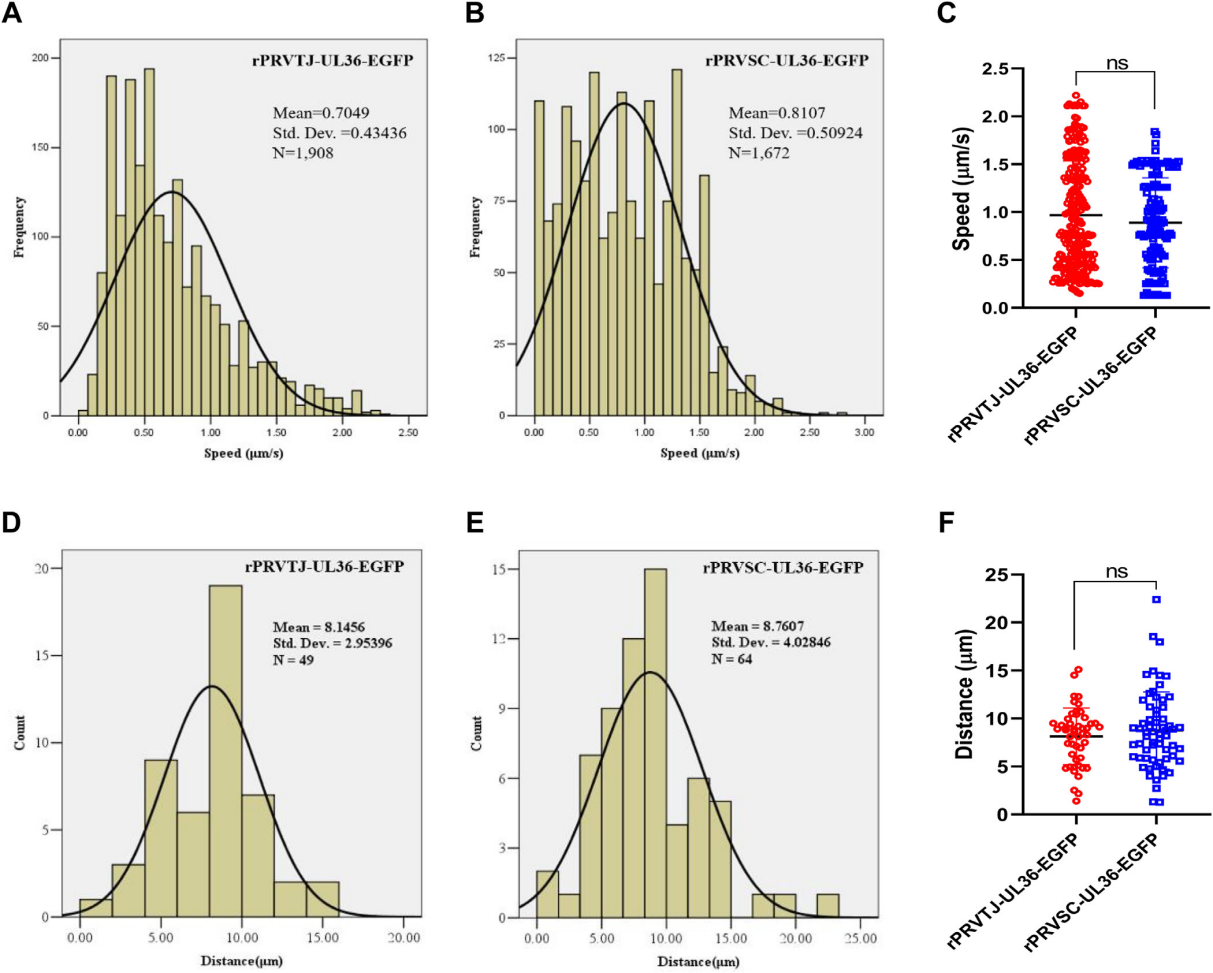
**Motion characteristics of the PRV TJ and SC strains in primary neuronal axons.** The neurons were initially seeded in the S compartment of microfluidics equipment and cultured for 3 to 4 days until the axons extended through the middle channel and reached the N compartment. Next, 10^6^ PFU of rPRVTJ-UL36-EGFP or rPRVSC-UL36-EGFP were inoculated into the N compartment, and the fluorescently marked viral particles in the channel were captured by time-lapse imaging. Simultaneously, their moving characteristics were analyzed. The instantaneous velocity distributions of retrogradely moving capsids for rPRVTJ-UL36-EGFP (*A*) and rPRVSC-UL36-EGFP (*B*) are presented. *C*, comparing the average speed of two strains using a *t* test. The total displacement of rPRVTJ-UL36-EGFP (*D*) and rPRVSC-UL36-EGFP (*E*) over a period of 10 s was quantified. *F*, *t* test to compare the average distance between two strains in 10-s intervals. The bars represent the means ± SD for more than 10 viral particles; ns, not significant, and *p* ≥ 0.05. EGFP, enhanced GFP; PFU, plaque-forming unit; PRV, pseudorabies virus.

Subsequently, the instantaneous velocity and displacement of single viral particles were analyzed in the primary neurons. A confocal laser scanning microscope was used to capture a total of 1908 and 1672 single viral capsids from rPRVTJ-UL36-EGFP and rPRVSC-UL36-EGFP, respectively, in the axon to calculate the instantaneous velocities. The results showed that the average instantaneous velocities of rPRVTJ-UL36-EGFP and rPRVSC-UL36-EGFP were 0.7049 ± 0.43436 and 0.8107 ± 0.50924 *μ*m/s, respectively (Fig. 4, *A* and *B*). Furthermore, the *p*-value for the *t* test assessing the difference in mean velocity was calculated as 0.1235 (Fig. 4*C*). The average displacements of rPRVTJ-UL36-EGFP and rPRVSC-UL36-EGFP within the 10-s interval were 8.1456 ± 2.95396 *μ*m from 49 viral particle movement events and 8.7607 ± 4.02896 *μ*m from 64 viral particle movement events, respectively (Fig. 4, *D* and *E*). The *p*-value calculated for the *t* test assessing on the mean distance in the 10-s interval was 0.3702 (Fig. 4*F*). These results collectively indicate that there is no significant difference in terms of instantaneous velocity or displacement between rPRVTJ-UL36-EGFP and rPRVSC-UL36-EGFP.

### The replication capacities of the PRV TJ and SC strains are similar in N2a cells

**Figure 5:**
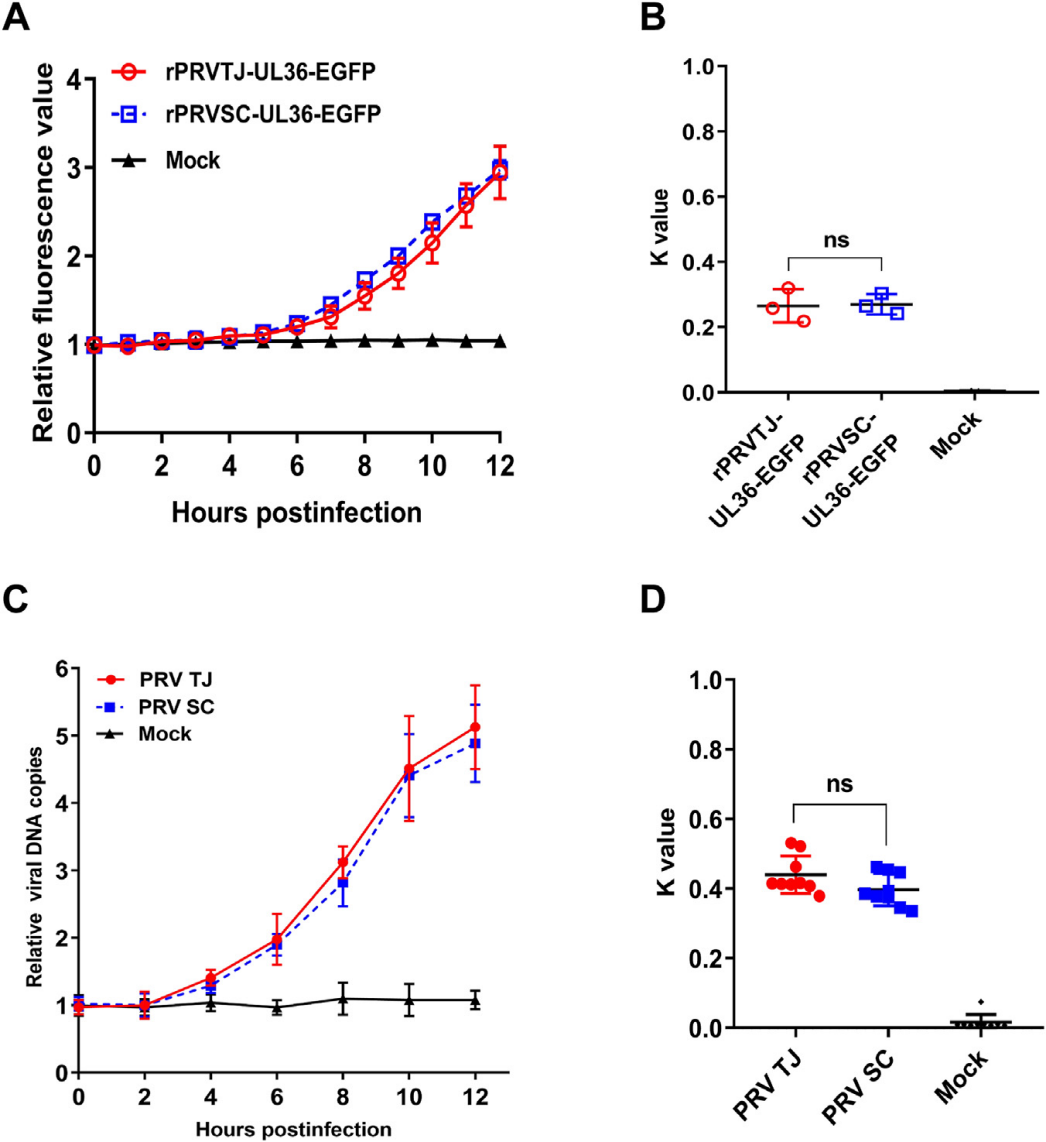
**Varied replication efficiency of the PRV TJ and SC strains in N2a cells.** N2a cells were infected with PRV TJ, PRV SC, rPRVTJ-UL36-EGFP, or rPRVSC-UL36-EGFP at 4 °C and washed 3 times with PBS, and another group was then transferred to 37 °C and cultured for 12 h. *A*, the change in fluorescence intensity of cells infected with rPRVTJ-UL36-EGFP and rPRVSC-UL36-EGFP from 0 to 12 hpi was measured. The fluorescence intensity of the single cells was captured every hour, with 0 hpi as the baseline. The absolute fluorescence intensity of each time point was divided by the baseline to obtain the relative fluorescence intensity. *B*, *K* values of each kinetic curve were calculated based on the increment of fluorescence intensity divided by the time interval. *C*, the change in viral DNA copies of PRV TJ and PRV SC from 0 to 12 hpi was measured. The viral DNA copies were quantified at 0 hpi as the baseline. The total viral DNA copies of each time point were divided by the baseline to obtain the relative viral DNA copies. *D*, the *K* values of each kinetic curve were calculated based on the increment of viral DNA copies divided by the time interval. The results were analyzed and the bars represent the means ± SD for three independent experiments; ns, not significant. EGFP, enhanced GFP; PRV, pseudorabies virus.

Although the invasion efficiencies of the PRV TJ and PRV SC strains differ, their respective replication capacities remain unknown. Given the variability in invasion efficiency between PRV TJ and SC, it becomes evident that the absolute viral DNA copies alone may not serve as a direct indicator of replication ability. Therefore, the relative replication efficiency of PRV was analyzed. The replication process of rPRVTJ-UL36-EGFP and rPRVSC-UL36-EGFP in N2a cells was monitored using laser scanning confocal microscopy in a 3-dimensional model from 0 to 12 hpi. Fluorescence signals were captured at 1-h intervals and the fluorescence intensity of a single cell was analyzed using the ZEISS ZEN microscopy software. The fluorescence intensity at 0 hpi was considered as the base value for evaluating the fluorescence intensity variation kinetics at each time point (35). The generated images revealed dynamic changes in gene replication across all the observed time points, with the fluorescence intensity remaining nearly unchanged at 0 to 4 hpi but subsequently experiencing rapid increments at 7 to 12 hpi (Fig. 5*A*). The curves based on the fluorescence intensity of rPRVTJ-UL36-EGFP and rPRVSC-UL36-EGFP at each time point shared similar trends. In addition, the K value of those curves was also analyzed based on the average relative increment in fluorescence intensity divided by the corresponding adjacent time intervals (Fig. 5*B*). We also examined the dynamics of gene copy number changes during the replication cycle of different PRV strains. N2a cells were inoculated with PRV TJ or SC at an MOI of 1, and the cells were collected at 2-h intervals following infection. The relative viral DNA copies were analyzed at each time point. The results showed that PRV TJ and SC exhibited similar kinetics (Fig. 5*C*). Furthermore, the K value was determined using the previously described method and demonstrated a similar distribution (Fig. 5*D*), indicating that PRV TJ and SC possess comparable genome replication efficiencies.

### The gB, gC, and gD of PRV TJ bind to their corresponding receptors more effectively than do those of PRV SC

**Figure 6:**
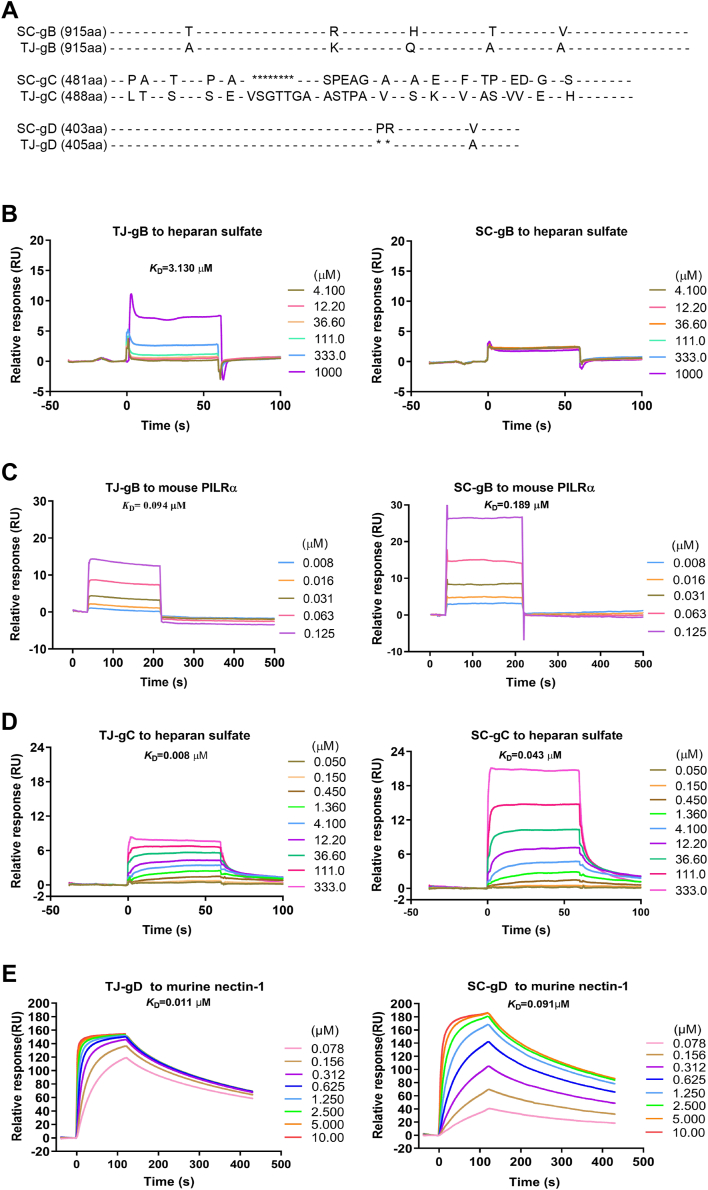
**The gB, gC, and gD proteins of the PRV TJ and SC strains interact with corresponding receptors.***A*, the schematic representation and alignment of gB, gC, and gD proteins of PRV TJ and SC strains are shown. "-" and "∗" indicate the consensus and missing sequences, respectively, while capital letters represent divergence sequences. *B*, SPR assay of different concentrations from 4.1 to 1000 *μ*M of heparan sulfate binding to immobilized TJ-gB (*left*) or SC-gB (*right*). *C*, SPR assay of different concentrations from 0.008 to 0.125 *μ*M of murine PILRα binding to immobilized TJ-gC (*left*) and SC-gC (*right*) in a dose-dependent manner. *D*, SPR assay of different concentrations from 0.05 to 333 *μ*M of heparan sulfate binding to immobilized TJ-gC (*left*) and SC-gC (*right*) in a dose-dependent manner. *E*, the SPR assay of different concentrations from 0.078 to 10 *μ*M of TJ-gD (*left*) or SC-gD (*right*) binding to immobilized mouse nectin-1 in a dose-dependent manner. gB, glycoprotein B; gC, glycoprotein C; gD, glycoprotein D; PRV, pseudorabies virus; SPR, surface plasmon resonance.

**Fig. S2:**
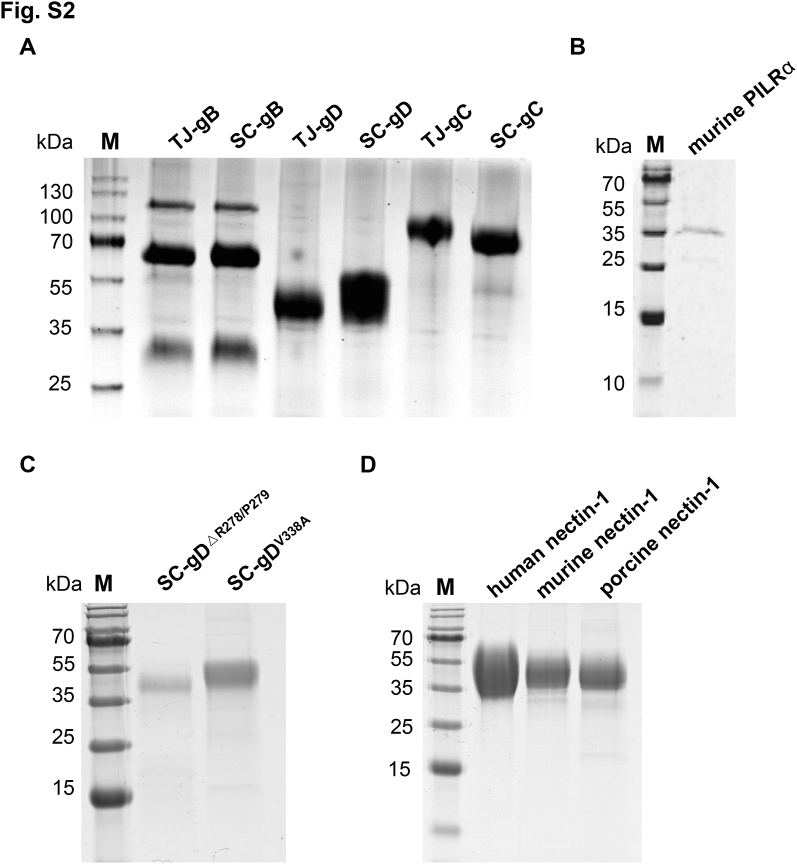
Images of SDS-PAGE for all the proteins used in the SPR experiments. All the recombinant proteins were produced, purified, and verified using SDS-PAGE followed by Coomassie blue staining. The images include (*A*) recombinant TJ-gB, SC-gB, TJ-gC, SC-gC, TJ-gD, and SC-gD; (*B*) recombinant murine PILRα; (*C*) recombinant SC-gD^ΔR278/P279^ and SC-gD^V338A^; and (*D*) recombinant human nectin-1, murine nectin-1, and porcine nectin-1.

The results presented above revealed that PRV TJ exhibits a higher binding affinity to N2a cells than PRV SC. It is well-known that the gB, gC, and gD of PRV play pivotal roles in recognizing and binding host cell receptors (18, 19, 21). To investigate the potential mechanism underlying the difference in binding affinity between PRV TJ and SC, the amino acid sequences of gB, gC, and gD were aligned, revealing multiple mutations between the two strains (Fig. 6*A*). Consequently, we speculated that these mutations might exert an influence on the receptor affinity of the viral proteins. To examine this hypothesis, surface plasmon resonance (SPR) technology was used to analyze the affinity of the gB, gC, and gD proteins with their respective receptors. Prior to SPR analysis, all the proteins were verified by SDS-PAGE (Fig. S2).

Initially, our results uncover that TJ-gB binds to heparan sulfate but with a relatively high *K*_*D*_ value of 3.13 *μ*M. However, no detectable signal was observed when SC-gB was incubated with heparan sulfate (Fig. 6*B*). These results suggest the presence of nonspecific binding between gB and heparan sulfate. Subsequently, we explored the interaction of gB with another receptor, murine PILRα. In this case, we observed a distinct and specific interaction when PILRα was introduced over both immobilized TJ-gB and SC-gB, with *K*_*D*_ values of 0.094 *μ*M and 0.189 *μ*M, respectively (Fig. 6*C*). We also detected heparan sulfate binding to gC, and the *K*_*D*_ values for TJ-gC and SC-gC were 0.008 and 0.043 *μ*M, respectively (Fig. 6*D*). Moreover, the *K*_D_ values for TJ-gD and SC-gD binding to the murine nectin-1 were 0.011 and 0.091 *μ*M, respectively (Fig. 6*E*). These results suggest that TJ-gB, TJ-gC, and TJ-gD had a higher affinity to their respective receptors than SC-gB, SC-gC, and SC-gD.

### The variations on the gB, gC, and gD proteins lead to enhanced viral entry to N2a cells

**Figure 7:**
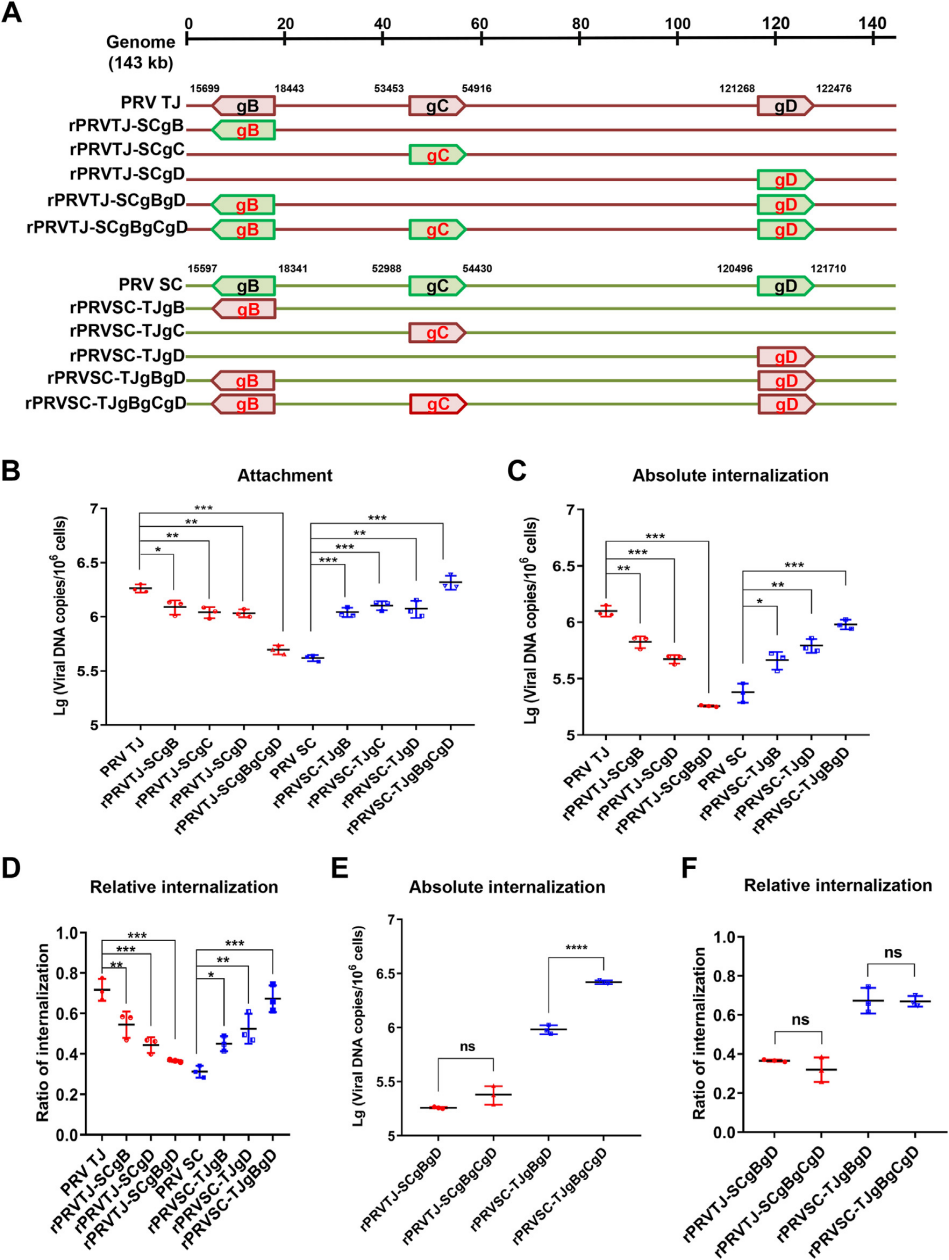
**The variations in the PRV gB, gC, and gD proteins affect virus entry into N2a cells.***A*, schematic model illustrating the recombinant PRV TJ or SC strains with mutations in the gB, gC, and gD of the PRV SC or TJ strain. The numbers indicate the gene location in the PRV genome. *B*, comparison of the attachment of the chimeric recombinant PRV with the single or triple gB, gC, and gD genes interchanged *versus* the parental viruses to N2a cells. *C*, absolute and (*D*) relative internalization of the chimeric recombinant PRV with the single or double gB and gD genes interchanged *versus* the parental viruses into N2a cells. *E*, absolute internalization and (*F*) relative internalization of rPRVTJ-SCgBgD, rPRVTJ-SCgBgCgD, rPRVSC-TJgBgD, and rPRVSC-TJgBgCgD in N2a cells. Bars represent the means ± SD for three independent experiments; ns, not significant; ∗*p* < 0.05; ∗∗*p* < 0.01; ∗∗∗*p* < 0.001. gB, glycoprotein B; gC, glycoprotein C; gD, glycoprotein D; PRV, pseudorabies virus.

To further investigate the contribution of each envelope glycoprotein to the increased invasion ability of PRV TJ, chimeric viruses with the gB, gC, and gD genes of PRV TJ and SC interchanged were constructed, as shown in Figure 7*A*. The adsorption efficiency of the single and three gene-interchanged chimeric viruses was compared with that of their parental viruses on N2a cells. As shown in Figure 7*B*, the single or triple substitution of the TJ gB, gC, and gD genes with the counterparts of the SC strain reduced viral adsorption, while the substitution of the gB, gC, gD single, and three genes of SC strain by gB, gC, gD single and three genes of TJ strain enhanced viral adsorption. Furthermore, the three gene-interchanged chimeric viruses induce a more dramatic change in absorption than any single gene-interchanged chimeric viruses. Collectively, these results indicated that variations in gB, gC, and gD genes synergistically contribute to the enhanced adsorption ability of the PRV TJ strain. Consequently, it becomes evident that all the three envelope glycoproteins contribute to the increased invasion ability of PRV TJ.

To further investigate the impact of gB and gD substitutions on PRV TJ and SC internalization, the absolute and relative internalization efficiency of chimeric PRV TJ and SC with gB or/and gD substituted were further analyzed (Fig. 7, *C* and *D*). As expected, the absolute and relative internalization efficiency of the chimeric PRV TJ with gB and gD substitutions, a single gene interchanged, was significantly lower than that of PRV TJ but still higher than that of PRV SC in the N2a cells. Double substitution of the gB and gD genes reduced the internalization efficiency of chimeric PRV TJ more significantly than any single substitution. Conversely, in the case of the SC strain, single or double substitution of gB and gD in the SC strain led to an enhancement in its internalization in the N2a cells. Interestingly, it was observed that mutations of 278th, 279th and 338th in the gD protein had a more significant effects on the internalization efficiency than mutations 82nd, 343rd, 447th, 634th, and 775th in the gB protein.

As variations in the amino acid sequence of gC might affect the internalization efficiency of the PRV TJ strain, we analyzed the absolute and relative internalization efficiency of the parental and chimeric PRV TJ and SC with gB/gD double or gB/gC/gD triple substitutions (Fig. 7, *E* and *F*). Notably, the viral DNA copies of rPRVSC-TJgBgCgD–infected cells were 2.7-fold higher than those of rPRVSC-TJgBgD–infected cells. However, there was no difference in internalization rate between the two chimeric strains. Furthermore, there was no difference in the absolute and relative internalization efficiency of the rPRVTJ-SCgBgD– or rPRVTJ-SCgBgCgD–infected cells.

The presented results suggest that the mutations in the gB, gC, and gD of PRV TJ altered the viral receptor affinity and contributed to the enhanced invasion efficiency of the virus into N2a cells. Further experiments using the chimeric viruses with the gB, gC, and gD genes of PRV TJ and SC interchange confirmed that these gene variations synergistically enhanced the adsorption ability of the PRV TJ strain and gD played a major role in this process.

### gD^ΔR278/P279^ and gD^V338A^ enhanced PRV entry into N2a cells

**Figure 8:**
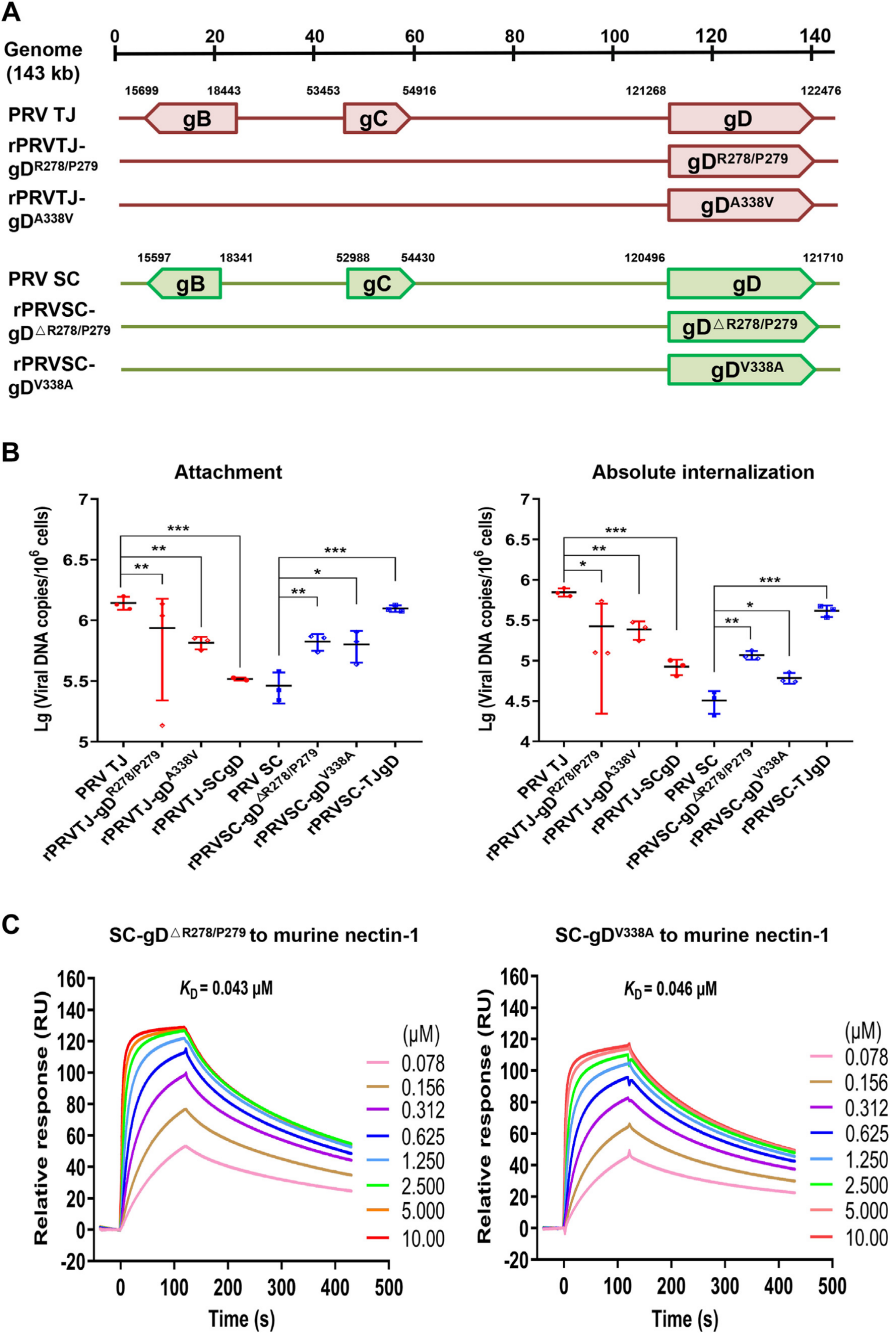
**SC-gD**^**ΔR278/P279**^**and SC-gD**^**V338A**^**enhanced PRV entry into N2a cells.***A*, schematic diagram showing the recombinant PRV TJ or SC strains with gD mutated. The SC-gD with the 278th and 279th residues deleted was named SC-gD^ΔR278/P279^, while SC-gD with the V338A mutation as SC-gD^V338A^. *B*, the attachment to (*left* plot) and internalization into (*right* plot) N2a cells of the chimeric recombinant PRVs with the single/double mutation residues interchanged *versus* the parental viruses. *C*, SPR assay of different concentrations (0.078–10.00 *μ*M) of the murine nectin-1 binding to immobilized SC-gD^ΔR278/P279^ (*left* plot) and SC-gD^V338A^ (*left* plot) in a dose-dependent manner. Bars represent the means ± SD for three independent experiments; ns, not significant; ∗*p* < 0.05; ∗∗*p* < 0.01; ∗∗∗*p* < 0.001. gD, glycoprotein D; PRV, pseudorabies virus; SPR, surface plasmon resonance.

To further elaborate on the key mutation residues of gD, chimeric viruses with the gD of PRV TJ and SC interchange were constructed, as shown in Figure 8*A*. The results showed that both the attachment and internalization efficiency of rPRVTJ-gD^R278/P279^ and rPRVTJ-gD^A338V^ were lower than those of PRV TJ but higher than those of rPRVTJ-SCgD (Fig. 8*B*). Similarly, both the attachment and internalization efficiency of rPRVSC-gD^ΔR278/P279^ and rPRVSC-D^V338A^ were higher than PRV SC but lower than those of rPRVSC-TJgD. Furthermore, the SPR results showed that the *K*_D_ values of SC-gD^ΔR278/P279^ and SC-gD^V338A^ were 0.043 and 0.046 *μ*M, respectively. This suggests that both SC-gD^ΔR278/P279^ and SC-gD^V338A^ bind the murine nectin-1 stronger than SC-gD (Fig. 8*C*). In summary, these findings indicate that the R278/P279 and V338 residues of gD are critical for efficient entry of PRV TJ.

### The TJ-gD protein binds the human and porcine nectin-1 receptors more effectively than the SC-gD protein

**Figure 9:**
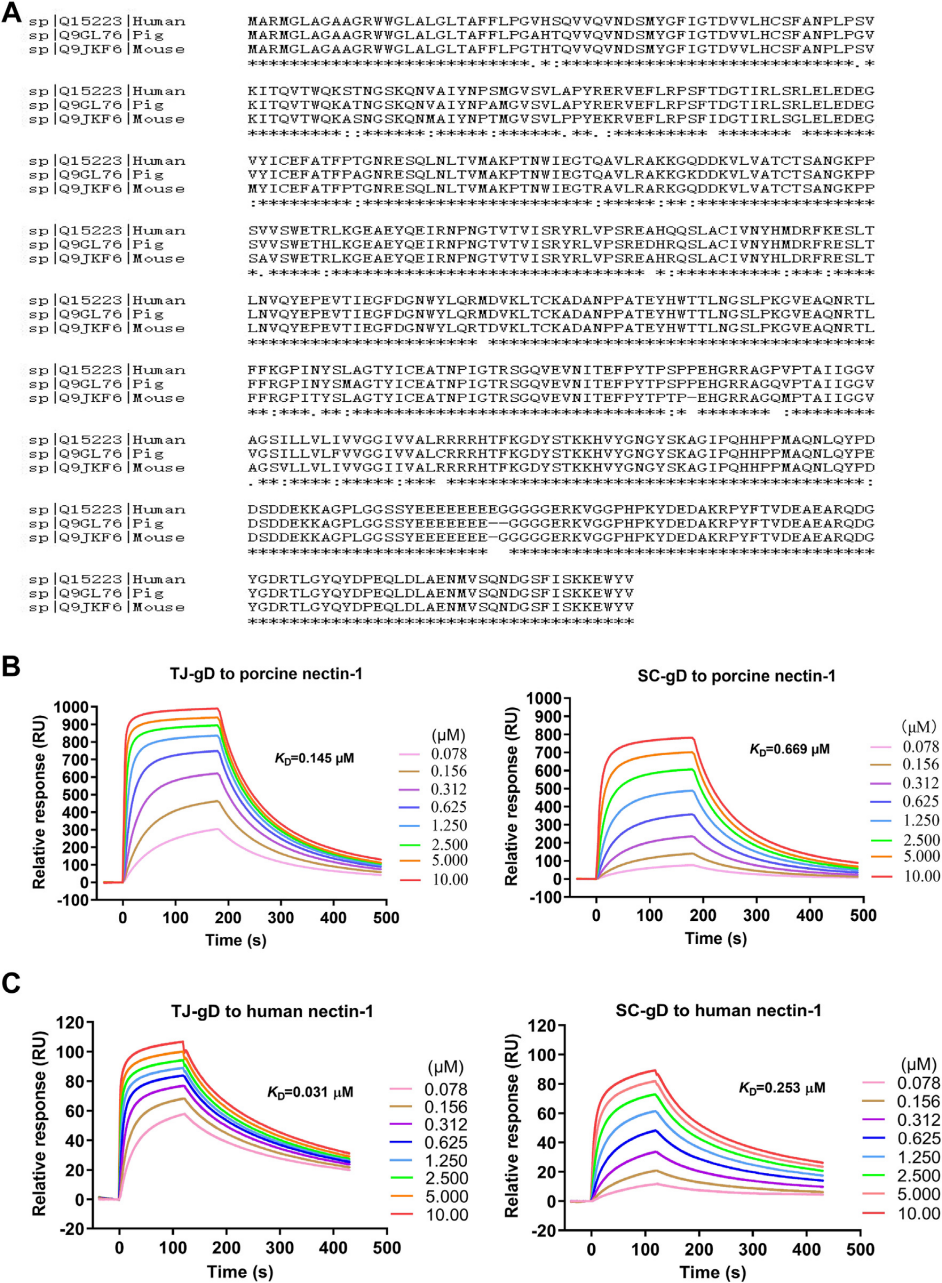
**The gD proteins of the PRV TJ and SC strains interact with the human and porcine nectin-1 receptors.***A*, multiple sequence alignment of the human, porcine, and murine nectin-1 using CLUSTAL 2.1 is presented. The SPR assay was performed to determine the binding of different concentrations (0.078–10 μM) of human nectin-1 (*B*) and porcine nectin-1 (*C*) to immobilized TJ-gD (*left*) and SC-gD (*right*) in a dose-dependent manner. gD, glycoprotein D; PRV, pseudorabies virus; SPR, surface plasmon resonance.

Although PRV is primarily a pig pathogen, there have been occasional reports of PRV isolation from humans (5). A sequence alignment of the nectin-1 proteins from mice, humans, and pigs revealed that 30 amino acid sites that differed between murine and human or porcine nectin-1, 19 amino acid sites that differed between the human and porcine nectin-1 (Fig. 9*A*). To assess the binding affinity of TJ-gD and SC-gD proteins to human and porcine nectin-1 receptors, SPR experiments were conducted alongside murine nectin-1. The results showed that the affinity of TJ-gD to porcine nectin-1 was higher than that of SC-gC, with *K*_D_ values of 0.145 and 0.669 *μ*M, respectively (Fig. 9*B*). Additionally, the *K*_D_ values for TJ-gD and SC-gD binding to human nectin-1 were 0.031 and 0.253 *μ*M, respectively (Fig. 9*C*). These findings indicate that TJ-gD had a higher affinity to both porcine and human nectin-1 receptors than SC-gD.

### The PRV TJ gB, gC, and gD proteins are associated with neurotropism, but not with virulence in mice

**Figure 10:**
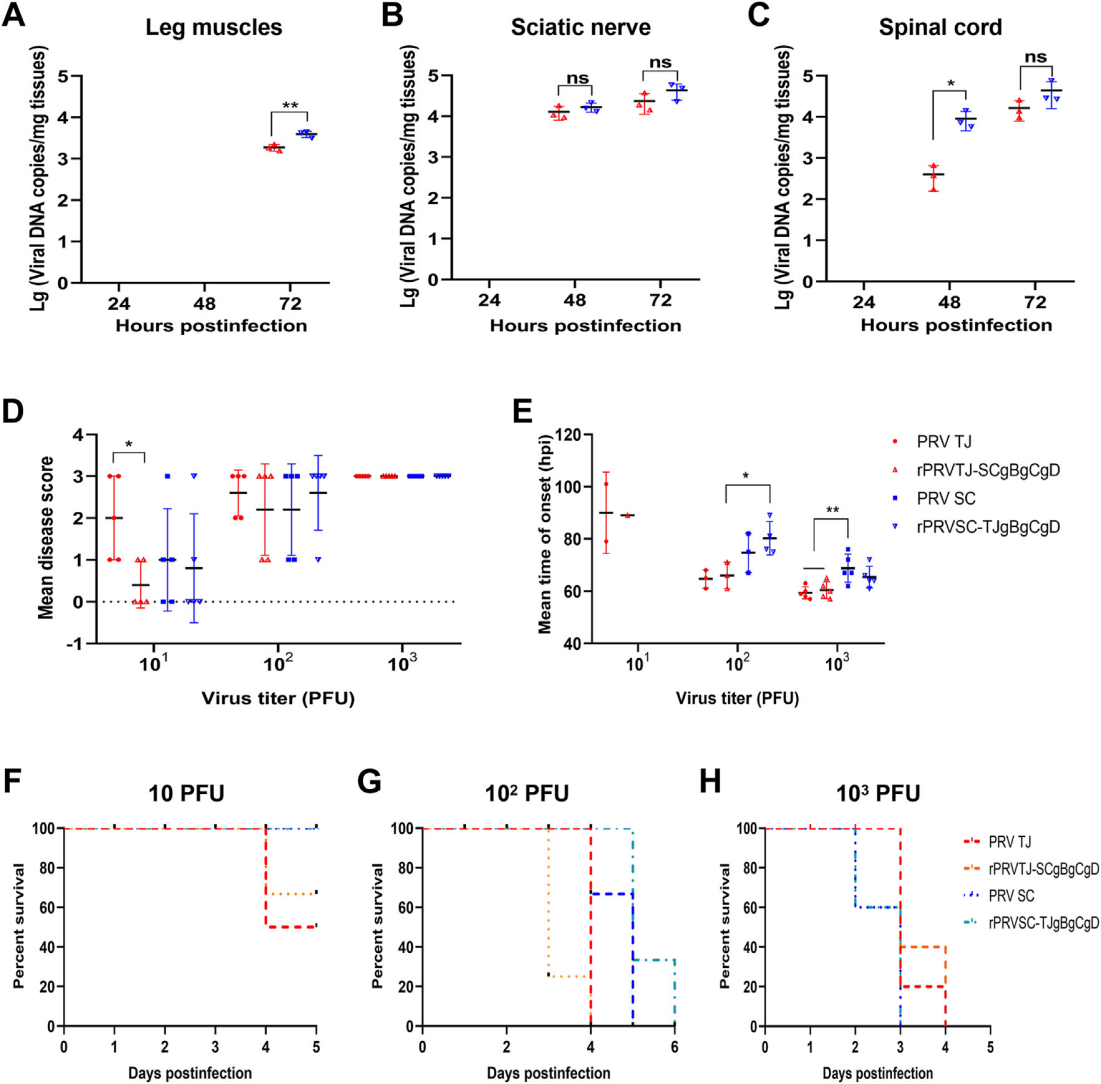
**Viral loads in the tissues of the mice infected with rPRVTJ-SCgBgCgD, rPRVSC-TJgBgCgD, or their parental viruses.** Mice were either mock-infected or infected with 10^3^ PFU of rPRVTJ-SCgBgCgD, rPRVSC-TJgBgCgD, PRV TJ, and PRV SC strains, and the tissue samples were collected at 24, 48, and 72 hpi from the legs (*A*), sciatic nerves (*B*), and spinal columns (*C*) on the inoculated side for the total DNA extraction. Viral DNA copies per microgram of tissue were quantified by using qPCR with specific primers targeting the *gI* gene. Mice infected with 10^1^, 10^2^, 10^3^ PFU of PRV TJ, PRVTJ-SCgBgCgD, rPRVSC-TJgBgCgD, or PRV SC strain were monitored daily for clinical symptoms, and the data were statistically analyzed to determine the mean disease score (*D*), mean time of onset (*E*), mean time of death, and mortality for 10^1^ PFU (*F*), 10^2^ PFU (*G*), 10^3^ PFU (*H*). The bars represent the means ± SD for three independent experiments; ns, not significant; ∗*p* < 0.05; ∗∗*p* < 0.01. PFU, plaque-forming unit; PRV, pseudorabies virus; qPCR, quantitative PCR.

To investigate the role of the gB, gC, and gD proteins in promoting the invasion efficiency of PRV TJ into nervous tissue *in vivo*, a group of 6-week-old SPF mice (*n* = 5) was i.m. infected with 10^3^ PFU of either rPRVTJ-SCgBgCgD or rPRVSC-TJgBgCgD in the left hind leg muscles. The tissues from the leg muscles, sciatic nerve, spinal cord, and brain were collected from all the mice at 24, 48, and 72 hpi to determine the viral load by qPCR. The viral loads in all four tissues increased over time, but there were differences in some tissues of two PRV strains–infected groups at certain time points. Specifically, the viral DNA copies in the leg muscles of the rPRVSC-TJgBgCgD–infected group were higher than those of rPRVTJ-SCgBgCgD at 48 and 72 hpi (Fig. 10*A*). However, there was no significant difference in the viral DNA copies in the sciatic nerves of the two groups at 48 and 72 hpi (Fig. 10*B*). The viral DNA copies in the spinal cord of the PRVSC-TJgBgCgD–infected group were higher than those of rPRVTJ-SCgBgCgD at 48 hpi, but no significant difference was detected at 72 hpi (Fig. 10*C*). No viral DNA was detected in the brains of either infected groups at 72 hpi, and none was detected at other time points.

The pathogenicity of the different PRV strains was evaluated in mice (Table 1). All the mock-infected mice remained healthy during the experiment. Disease score, mortality, time of itching onset and death were statistically analyzed (Fig. 10, *D*–*G*). The mean disease score in the 10 PFU rPRVTJ-SCgBgCgD–infected group was significantly lower than that in the PRV TJ group (Fig. 10*D*). The mean time of itching onset in the 10^3^ PFU rPRVSC-TJgBgCgD–infected group was significantly higher than that in the PRV TJ and rPRVTJ-SCgBgCgD–infected groups. Meanwhile, the 10^2^ PFU rPRVSC-TJgBgCgD–infected group was significantly higher than the rPRVTJ-SCgBgCgD–infected groups (Fig. 10*E*). There was no significant difference observed in the mean time of death between most of the groups except for the 10^2^ PFU of rPRVTJ-SCgBgCgD– and rPRVSC-TJgBgCgD–infected groups (Fig. 10, *F*–*H*). All the mice in the four 10^3^ PFU PRV-infected groups died (Fig. 10*H*), while most mice in the 10^2^ PFU PRV-infected group exhibited clinical signs, and the mortality was between 60 to 80% (Fig. 10*G*). Only two mice in the 10 PFU PRV TJ or one in the rPRVSC-TJgBgCgD–infected groups died (Fig. 10*F*). To determine the median lethal doses (LD_50_) of each PRV strain, the number of deaths within different challenge doses was calculated. The LD_50_ of rPRVTJ-SCgBgCgD (10^1.83^ PFU) was the same as PRV SC, while the LD_50_ of rPRVSC-TJgBgCgD (10^1.5^ PFU) was the same as PRV TJ. These results suggest that the gB/gC/gD triple substitutions had a minor impact on the virulence of PRV TJ or PRV SC.

## Discussion

In this study, we investigated the distribution of the virus in various organ tissues following infection with PRV TJ and SC strains in mice. Both strains were found to be predominantly present in peripheral nerves, such as perivascular nerves, sciatic nerves, and some myelinated neurons outside of the spinal cord. Lower viral loads were detected in the brain and kidneys, and no virus was detected in the heart, liver, spleen, and lungs. These results demonstrate that PRV can efficiently invade the nervous system through the neuromuscular junction, as previously reported for its invasion of the primary inoculated site and spread through the synaptic junction and retrograde transport along the vagal nerve to the brain (36). The viral load in the various tissues of PRV-infected mice varied depending on the inoculation route. For instance, intranasal inoculation resulted in the highest viral load in the trigeminal ganglia, while intramuscular injection led to the highest titer in the spinal cord (37). Moreover, when the challenge dose exceeded 10^3^ TCID_50_, PRV was detected in the spleen, liver, and kidneys following intramuscular infection routes (37).

Furthermore, despite the significant accumulation of viral antigens in myelinated neurons near the spinal cord, the viral antigens in the spinal cord remained undetectable through IHC. However, high levels of viral genome were detected in spinal samples by qPCR. This discrepancy suggests the existence of a barrier between peripheral and central neurons, which may impede the rapid entry of the virus into the central nervous system. Meanwhile, the host might also utilize the barrier to resist virus entry (38). Dramatically, the viral loads in the brain were below the qPCR detection threshold at 2 days postinfection, while the necrosis of some neuron and Purkinje cells in the brain appeared simultaneously. This suggests that the interaction between PRV and the host nervous system occurs independently of local virus reproduction.

In this study, we compared PRV TJ and SC strains in the axonal transport and replication stages. It is reported that the UL36, UL37, and US3 proteins cotransported with the capsid particles as they traversed along the axons towards the cell body. Notably, UL36 protein was found to directly bind to the dynein subunit DIC, as well as to the dynein-activating protein subunits p150 and p50 (39, 40, 41). Despite the presence of partial amino acid sequence variations in UL36, UL37, and US3 proteins between PRV TJ and SC strains, the average velocity of axonal retrograde mobility within nerve axons did not exhibit a significant difference. It is important to highlight, however, that lysine ubiquitination at position 442 of UL36 led to a substantial reduction in anterograde axonal transport velocity (42). This suggests that the impact of genetic variants on anterograde axonal transport warrants further investigation. Additionally, the catalytic subunit of PRV DNA polymerase, UL30, displayed no discernible differences between the two strains. Furthermore, the replication efficiency of both viruses remained unaffected by the partial genetic disparities observed in the accessory subunit UL42 (43).

It was confirmed that the invasion efficiency of PRV TJ to nerve cells was higher than that of PRV SC. Moreover, the mutations in the gB, gC and gD were all responsible for enhancing the attachment of PRV TJ to the nerve cells. Our results reveal that the *K*_*D*_ of PILRα to SC-gB was 2-fold higher than TJ-gB; thus, the afﬁnity of PILRα binding to SC-gB was lower than that of TJ-gB, but no specific binding was found between TJ-gB/SC-gB and heparan sulfate. There are five distinct arginine amino acids between TJ-gB and SC-gB. These unique residues within the TJ-gB protein may enhance its binding to the PILRα receptor. Notably, the gB protein of PRV can interact with both PILRα and PILRβ receptors, whereas the gB protein of HSV-1 can only interact with PILRα but not PILRβ receptor (44).

Moreover, it has been reported that the PRV gB protein only bound strongly to heparan sulfate in the presence of gC by heparin affinity chromatography (45). In our study, gB alone did not exhibit specific binding to heparan sulfate when assessed using SPR technology. In contrast, the binding of the HSV gB protein to heparan sulfate did not depend on gC (46, 47, 48). Additionally, the residues Trp187, Tyr192, Phe275, and Tyr276 of PRV gB were observed to interact liposomes and form a fusion loop (49). The mutation of residue Asn735 to Ser in the PRV gB protein has been shown to facilitate virus entry independently of gH/gL (50). The five differential sites between the TJ-gB and SC-gB proteins located at amino acid positions 82, 343, 447, 634, and 775. Further investigation is required to ascertain whether these five differential residues have an impact on fusion or their binding to other gB receptors.

The experiments also detected the binding ability of gC to heparan sulfate by SPR, and we found that the *K*_*D*_ of heparan sulfate to SC-gC was 5-fold higher than TJ-gC and the affinity of heparan sulfate to TJ-gC was higher than that of heparan sulfate to SC-gC. The diverse amino acid residues between TJ-gC and SC-gC are primarily located at the N terminal 14 to 243 amino acids of the gC protein, which contain three heparan-binding domains (HBDs) of PRV-Becker strain gC. HBD1 (amino acid positions 75–82) was found to bind heparin with at least 10-fold greater afﬁnity than HBD3 (amino acid residues 135–140) (51). Therefore, it was reasonable to conclude that amino acid mutation in the N terminal of gC enhanced the affinity of heparan sulfate to TJ-gC.

The crucial step for PRV entry into cells involves gD binding to the nectin-1 receptor, which is responsible for attachment and internalization. According to the SPR results, the *K*_D_ values of murine and human nectin-1s were found to be 13-fold and 5-fold lower than that of the porcine nectin-1, respectively. The same trends were detected in TJ-gD and SC-gD proteins binding to the three nectin-1 receptors. These results were consistent with previous reports, which found that the *K*_D_ values of PRV gD binding to the human and porcine nectin-1 were 0.191 and 0.301 *μ*M, respectively (20). Therefore, the results illustrated that PRV gD exhibited a tighter association with the human and murine nectin-1s than with the porcine nectin-1.

Based on the crystal structural analysis of the porcine nectin-1–gD complex, the major receptor-engagement components of gD include its N-loop, the *α*1′ helix in the IgV core, and helices *α*2′, *α*3′, *α*3, and the *α*3'/*α*3 loop in its C-terminal extension (20). The ectodomain of gD includes amino acid residues 1-337, the amino acid residues 201-219 formed *α*3’/*α*3 loop, and the large C-terminal polypeptide after the 219th residue did not bind to the nectin-1 receptor directly. The C-terminal residues 261-316 were important for triggering membrane fusion (52).

In this study, the affinity of TJ-gD, SC-gD, SC-gD^△R278/P279^, and SC-gD^V338A^ proteins to murine nectin-1 was detected using SPR. The *K*_D_ values of the two mutation proteins were approximately 4-fold higher than that of TJ-gD and 5-fold lower than that of SC-gD. Similar results were reported that the affinity of PRV gD^1-284^ (a C-terminus–truncated gD variant) for nectin-1 was approximately 12 to 16 folds higher than that of gD^1-337^ (the whole ectodomain of gD using SPR technology (20)). The isoelectric points of SC-gD and TJ-gD are 9.566 and 9.472, respectively. Notably, the absence of R278 and P279 does not alter the isoelectric point. Upon introducing the V338A mutation at the 338th position of the protein, the isoelectric point of the mutant protein matches that of TJ-gD, which is 9.472. This suggests that the V338A mutation may modify the electrostatic attraction between gD and nectin-1. The interaction interface between the two proteins is influenced by the presence of R278 and P279, and their absence may lead to an enhancing in structural compatibility.

In addition to the changes in virus invasion efficiency detected by *in vitro* experiments, the mutations of gB, gC, and gD proteins affect the pathogenicity of PRV in mice in this research. The pathogenesis of PRV TJ and SC strains were evaluated and compared in mice and pigs by Luo *et al* (4). In mice, the LD_50_ of the TJ strain (10^2.3^ TCID_50_) was lower than that of the PRV SC strain (10^3.0^ TCID_50_), which is consistent with our results. Mouse, as a small-animal model, was very responsive to PRV compared with the pig, the natural host of PRV. In this study, 10 PFU of PRV TJ or rPRVSC-TJgBgCgD were lethal for mice. Although the LD_50_ is an index to evaluate the pathogenicity of these four PRV strains, the difference is not very significant. An obvious difference in clinical signs of PRV TJ and SC was itching (53). The indexes, including clinical signs, mean onset time, and mean death time, shown in Table 1, also reflected the changes induced by the substitution of gB, gC, and gD genes of PRV TJ and SC led in mice. The gB, gC, and gD proteins are also involved in other viral infection processes. gB binds paired PILR*β* and triggers NK cell-mediated lysis in both gB-transfected and PRV-infected cells (30). gC is involved in virus release with gE and gI and may help stabilize virus particles (54). Recombinant PRVs with gC, gE, or gI deletions were significantly attenuated (55).

The latest case report of human infection with PRV variant strains could infect human retinal epithelial cells more effectively than PK-15 cells. Infection with these strains also leads to severe neurological deficits, with some patients becoming vegetative (5), which suggests that the PRV variant strains show higher tropism to neurons than the previous PRV strains. We observed that the PRV TJ strain possesses a stronger tropism for the nervous system than the PRV SC strain.

In summary, our study revealed significant differences in viral invasion between PRV TJ and SC strains, with mutations in the gB, gC, and gD proteins increasing virus adsorption efficiency, while mutations in gB and gD increased virus internalization efficiency, with gD playing a key role. The mutated envelope protein had a stronger affinity for its receptor protein, which improved the efficiency of adsorption and membrane fusion. Our findings suggest that PRV has evolved towards greater neurotropism and higher pathogenicity, which highlights the importance of developing new strategies for the prevention and control of PRV infections.

## Experimental procedures

### Cells and virus strains

PK-15 (Porcine kidney cell line), N2a (Neuro-2a, mouse neuroblastoma cell line), and HEK293T (Human embryonic kidney cell line) were maintained in Dulbecco's modified Eagle medium (DMEM) supplemented with 10% fetal bovine serum, 100 U/ml penicillin, and 100 μg/ml streptomycin. HEK293S cell line was maintained in Pro293 serum-free media. The PRV TJ (GenBank no. KJ789182.1) and SC (GenBank no. KT809429.1) strains were propagated and titrated on PK-15 cells. The reporter viruses rPRVTJ-UL36-EGFP and rPRVSC-UL36-EGFP expressing EGFP fused with the UL36 protein were previously constructed and reported by our laboratory (33).

### Primary neuronal cultures and microfluidic chambers

DRGs were isolated from the spines of neonatal BALB/c mice, washed three times with PBS, then digested with 1 mg/ml collagenase/dispase (catalog no. 10269638001; Roche) at 37 °C for 45 to 60 min. The cells were neutralized with fetal bovine serum and all the DRGs cells were plated in the S chamber of a microfluidic device, which was then incubated overnight. To clear dividing cells, 5 nM cytosine arabinoside (AraC) (catalog no. C6645; Sigma) was added to the wells, and the medium was replaced daily. The microfluidics device was fixed onto a sterile glass coverslip that was treated with poly-DL-ornithine hydrobromide (catalog no. P8638; Sigma) overnight and laminin (catalog no. 23017015; Invitrogen) for at least 6 h before use (33). To perform retrograde transport analysis, PRV was added to the N chamber, and the viral titers in the N chamber medium were detected. Single viral particles moving in the axon were observed and captured using a confocal laser scanning microscope (Zeiss LSM 800) at 37 °C with 5% CO_2_.

### Real-time qPCR

The genome of the PRV-infected mouse tissues and cells was extracted using the MagaBio plus virus DNA purification kit (catalog no. BSC11S1C; BioFlux). Viral genome copies were quantified using primers and probes as described previously (33).

### Confocal immunofluorescence imaging

N2a cells were seeded and cultured in glass-bottom dishes overnight, then infected with PRV TJ and SC at an MOI of 100 and incubated for 2 h at 4 °C. The cells were fixed with 4% paraformaldehyde and washed with PBS three times. Subsequently, the viral gB protein and cytomembrane were stained with anti-gB monoclonal antibody (S81AEESLE87 as epitope) and the lipophilic carbocyanine fluorescent dye DiI (catalog no. D3911; Invitrogen), respectively. Finally, the cell nuclei were stained with DAPI, and images were captured by an LSM880-ZEISS confocal laser scanning microscope with Airyscan (Zeiss).

### Red/ET-mediated recombination

The fosmid libraries of the PRV TJ and SC strains developed previously in our lab were used to construct the recombinant chimeric PRV strains. The *gB*, *gC*, and *gD* genes of PRV TJ and SC were interchanged by Red/ET-mediated recombination technology according to the protocols of the Counter Selection BAC Modification Kit (catalog no. K002; Gene Bridges). The *gB*, *gC*, and *gD* genes, along with the selectable antibiotic cassette (*rpsL-neo)* flanked by 50-bp homology arms of *gB*, *gC*, and *gD*, were amplified by PCR using the primers listed in Table 2.

**Table 2:** Primers for the construction of gene-interchanged chimeric viruses

Names	Sequences (5′-3′)	Target gene
gB-rpsl-F1	GTGACGCGGGCCGCCTCGGCCTCGCCCGCGCCCGGGACGGGCGCCACCCCGGCCTGGTGATGATGGCGGGATCG	rpsL-*gB*1 arms
gB-rpsl-R1	CGCCTTCTTGACGAGTTCTTCTGAAGCTGCGCCAGCTCGTTCGAGATCAGCGGGCGGAAGGCCACCACGAAGCC	
gB-fos-F1	CATCGGCCCGGGCACCACGGCG	*gB*1 (40–1581 bp)
gB-fos-R1	GCCCGTGGTGATGCGCAGGTGC	
gB-rpsl-F2	CCTACGACCACATCCAGGCGCACGTGAACGACATGCTGAGCCGCATCGCGGGCCTGGTGATGATGGCGGGATCG	rpsL-*gB*2 arms
gB-rpsl-R2	CGCCTTCTTGACGAGTTCTTCTGACGTCGGGGTCCTCGTTCTCGAGGCGCTGGTAGTGCCGGCGGCGCGTGGCC	
gB-fos-F2	GAGCTCGAGCGCCTCGGCCTCG	*gB*2 (1438–2809 bp)
gB-fos-R2	ACGAGGCGGACGCGAGACGTGC	
gC-rpsl-F	AACCGCGATGGGGGGACGGGGGGCCATTCGCACGCGCCATGGCCTCGCTCGGCCTGGTGATGATGGCGGGATCG	rpsL-*gC* arms
gC-rpsl-R	GCACGCTGCGCCGCGGGTAGTAGTCGCGGACGACGCACACCGCCCGGAAGTCAGAAGAACTCGTCAAGAAGGCG	
gC-fos-F	TGCGCCACACCCGCGCGTACCG	*gC* (179–976 bp)
gC-fos-R	CCGCGTCGTCCGCGGCCGCGAG	
gD-rpsL-F	TGGAGCGACGACAGCTTCAAGCGGGGCGTGGACGTGATGCGATTCCTGACGGCCTGGTGATGATGGCGGGATCG	rpsL-*gD* arms
gD-rpsL-R	CCGGGTCACGTCGCGCGCCACCATCATCATCGACGCCGGTACTGCGGAGGTCAGAAGAACTCGTCAAGAAGGCG	
gD-fos-F	TGGAGCGACGACAGCTTCAAGCGGGGCGTGGACGTGATGCGATTCCTGACGCCGTTCTACCAGCAGCCCCCGCA	*gD* (616–1259 bp)
gD-fos-R	CCGGGTCACGTCGCGCGCCACCATCATCATCGACGCCGGTACTGCGGAGGCTACGGACCGGGCTGCGCTTTTAG	
gD-mut-F	TGCCCGAGCCGGCGACGCGGGACCACGCCG	*gD*_*M1*_/*gD*_*M2*_ (616–1259 bp)
gD-mut-R	CGGCGTGGTCCCGCGTCGCCGGCTCGGGCA	

### PRV binding and internalization assays

To determine PRV binding, 10^6^ cells were infected with PRV (MOI = 10) for 2 h at 4 °C, then the cells were washed three times with ice-cold PBS to remove the unbound PRVs and collected to determine the viral DNA copies of the attached PRV on the N2a cells. To determine PRV internalization, cells were transferred to 37 °C for 1 h and treated with 200 μl of 0.25% trypsin-EDTA at 4 °C for 15 min to remove the unbound PRVs (56). The total viral genomic DNA of the PRV-infected cells and the control cells were extracted, and the numbers of adsorbed and internalized PRV genomic were quantified by qPCR. The relative internalization ratio was obtained by dividing the numbers of internalized PRV by the number of adsorbed PRV.

### Flow cytometry

N2a cells were detached using 2 mM EDTA, and approximately, 10^5^ N2a cells were incubated with 10^7^ PFU of either PRV TJ or PRV SC (MOI = 100) for 2 h at 4 °C. After incubation, the cells were stained with an anti-gB monoclonal antibody for 2 h at 4 °C and then with goat anti-murine IgG (H+L) Alexa Fluor 488 for 45 min at 4 °C. A total of 10^4^ cells were analyzed by a BD Accuri C6 Plus Flow Cytometer (BD Bioscience), and the data were analyzed using the FlowJo software version 10 (TreeStar).

### Puriﬁcation of the recombinant proteins expressed in HEK293S cells

The coding sequences of the murine nectin-1 (residues 1–324), murine PILRα (residues 1–909), TJ-gB (residues 59–756), SC-gB (residues 59–756), TJ-gD (residues 1–353), SC-gD (residues 1–355), TJ-gC (residues 1–459), and SC-gC (residues 1–452) ectodomains were amplified by PCR using the primers listed in Table 3. A secreted signal peptide sequence was designed in the N terminus. The gB gene was fused with 6×His- and Strep (II) tag-coding sequences at the N terminus, while the other genes were fused at the C terminus. The resulting DNA fragments were cloned into *Eco*RI and *Xho*I sites of the pLVX-IRES-ZsGreen vector. The corresponding VSV-G–pseudotyped recombinant lentivirus was rescued, and HEK293S cell lines were transduced. The recombinant protein in the supernatant of the transduced cells were collected and purified using Ni^+^ or Strep-Tactin XT Sepharose column affinity chromatography (GE Healthcare) following standard protocols.

**Table 3:** Primers for the construction of recombinant plasmids

Names	Sequences (5′-3′)	Target gene
plvx-gB-SP-F	GTCCTGCATCATCCTGTTCCTGGTGGCCACCGCCACCGGCGTGCACTCCGGCGGCCACCACCACCACCACCACTCCGC	Signal peptide
plvx-gB-tag-R	GAGGCGGCCCGCGTCACGGCCGCTGCTGACTTCTCGAACTGGGGGTGGGACCAGGCGGAGTGGTGGTGGTGGTGGTGGCC	His and Strep-tag
plvx-gB-F	GAGAAGTCAGCAGCGGCCGTGACGCGGG	*gB* (175–2268 bp)
plvx-gB-R	GGAGGGAGAGGGGCGGGATCCTTACAGCAGCACCACGTTGTGGTCC	
plvx-gC-F	GTCCTGCATCATCCTGTTCCTGGTGGCCACCGCCACCGGCGTGCACTCCATGGCCTCGCTCGCGCGTGC	*gC* (76–1356 bp)
plvx-gC-R	GGAGGGAGAGGGGCGGGATCCTCAGGCGGAGTGGTGGTGGTGGTGGTGGCCGCCCATGCTGGTCACGACGGGCCAGCT	
plvx-gD-F	GTCCTGCATCATCCTGTTCCTGGTGGCCACCGCCACCGGCGTGCACTCCATGCTGCTCGCAGCGCTATTG	*gD* (1–1050 bp)
plvx-gD-R	GGAGGGAGAGGGGCGGGATCCTCAGGCGGAGTGGTGGTGGTGGTGGTGGCCGCCCGAGACGCCCGGCGCGGCGGTG	
Mou-Nectin-F	GTCCTGCATCATCCTGTTCCTGGTGGCCACCGCCACCGGCGTGCACTCCAACGACTCCATGTATGGCTTCAT	Mouse-Nectin1 (108–1062 bp)
Mou-Nectin-R	GGAGGGAGAGGGGCGGGATCCTCAGGCGGAGTGGTGGTGGTGGTGGTGGCCGCCGGCTGTGGGCATCTGCCCG	
pIRES-gB-F	ATAGGCTAGCCTCGAGAATTCGCCACCATGCCCGCTGGTGGCGGTCTTT	*gB* (1–2268 bp)
pIRES-gB-R	CATGCTCGACGCGTGAATTCCTAGTGGTGGTGGTGGTGGTGGCCGCCGGGGGCGTCGGGGTCCTCGTT	
pIRES-gD-F	GCCGCCCGGGTCGACTCTAGACTAAGCGTAATCTGGAACATCGTATGGGTAGCCGCCCGGACCGGGCTGCGCTTTTA	*gD* (1–1050 bp)
pIRES-gD-R	GCCGCCCGGGTCGACTCTAGACTAAGCGTAATCTGGAACATCGTATGGGTAGCCGCCCGGACCGGGCTGCGCTTTTA	
Por-Nectin-F	GTCCTGCATCATCCTGTTCCTGGTGGCCACCGCCACCGGCGTGCACTCCAG AACGACTCCATGTATGGTTTCA	*Por-Nectin* (1–963 bp)
Por-Nectin-R	GGAGGGAGAGGGGCGGGATCCTCAGGCGGAGTGGTGGTGGTGGTGGTGGCC ATGGCCGTGGGCACCTGCCCTG	
Hu-Nectin-F	GTCCTGCATCATCCTGTTCCTGGTGGCCACCGCCACCGGCGTGCACTCCAGACTCCATGTATGGCTTCATCG	*Hu-Nectin* (1–936 bp)
Hu-Nectin-R	GGAGGGAGAGGGGCGGGATCCTCAGGCGGAGTGGTGGTGGTGGTGGTGGCC CCTGGCTGCACTTCCCAGACCC	

### SPR measurements

The binding kinetics between the soluble gB, gC, gD, and their respective receptors were analyzed at 25 °C using a Biacore 8K machine with a Series S Sensor Chip CM5 (GE Healthcare). PBST buffer (2 mM KH_2_PO_4_, 8 mM Na_2_HPO_4_, 136 mM NaCl, 2.6 mM KCl, 0.5% Tween 20, pH 7.4) was used for all the measurements. The protein was immobilized on a Series S Sensor Chip CM5 (GE Healthcare Life Sciences) using 10 mM acetate buffer at a pH of 5.5, 5.0, or 4.5. The surface of the sensor chip was exposed to varying concentrations of ligand. A blank channel was used as a negative control. After data collection in each cycle, the sensor surface was regenerated with 10 mM NaOH. The protein-ligand binding affinity is evaluated by a dissociation constant (*K*_*D*_) values based on a concentration-dependent kinetic study using SPR technology. A smaller *K*_*D*_ value signifies a stronger binding affinity, while a *K*_*D*_ value exceeding 1 μM indicates nonspecific binding between the protein and ligand.

### Experimental infection of mice with PRV

The experimental protocols involving mice were approved by the Harbin Veterinary Research Institute Animal Welfare Committee (No. Heilongjiang-SYXK-200710-2). All the experiments were performed in a biosafety level II laboratory with strict biosecurity measures. Forty-five SPF 6-week-old female BALB/c mice were randomly divided into three groups, with 20 mice in the PRV TJ and SC groups, and five in the mock-infected group. The clinical signs of all the mice were recorded throughout the experiment. For each mouse, a total of 100 μl of filter-sterilized 10^3^ PFU PRV TJ or PRV SC suspended in DMEM was injected into the left hind limb muscle, and DMEM only was used as a control. To trace viral spreading, muscle tissue samples from the leg around the injection point, sciatic nerve, lumbar spinal cord, and brain were collected each day after the infection, and the clinical signs were recorded. A total of five samples per group per day were obtained. Tissue was bisected, with half kept in formaldehyde for H&E and IHC assays and the other half frozen at −80 °C for qPCR.

### Immunohistochemical and histopathological examinations

The leg, sciatic nerve, spinal cord, and brain tissues were collected from the mice and fixed with 10% formalin for 48 h, then transferred to 70% ethanol, embedded in paraffin wax. 5 *μ*m-thick sections were sliced and mounted on glass slides. The tissue sections were deparaffinized with xylene and rehydrated through a descending-graded ethanol series. Antigen retrieval was performed using a citrate buffer solution.

For immunohistochemical examination, the tissue sections were blocked with 10% normal goat serum and incubated with anti-gB antibodies overnight at 4 °C. After washing 3 times in TBST buffer, the sections were incubated with the SignalStain Boost IHC Detection Reagent (HRP, mouse) for 30 min at room temperature, followed by three washes with PBS buffer. The sections were then counterstained with hematoxylin. For the histopathological assay, the tissue sections were stained with H&E. Finally, the tissue sections stained with H&E or IHC were dehydrated with ethanol and xylene and then covered with a mounting medium for long-term storage.

### Statistical analysis

The results are presented as the mean ± SEM from at least three independent experiments. Differences between the two groups were determined by Student's two-tailed *t* test. All the statistics analyses were performed using GraphPad Prism version 8.

## Data availability

All data are contained within the article.

## Supporting information

This article contains supporting information (Figs. S1 and S2).

## Conflict of interest

The authors declare that they have no conflicts of interest with the contents of this article.
